# Seasonal and Spatial Variation in Dissolved Heavy Metals in Liaodong Bay, China

**DOI:** 10.3390/ijerph19010608

**Published:** 2022-01-05

**Authors:** Weijun Guo, Jibing Zou, Sihong Liu, Xuewen Chen, Xiangpeng Kong, Hong Zhang, Tiaojian Xu

**Affiliations:** 1 College of Environmental Sciences and Engineering, Dalian Maritime University, Dalian 116026, China; gwj5268@dlmu.edu.cn (W.G.); bing19818923922@dlmu.edu.cn (J.Z.); EminentLiu@outlook.com (S.L.); kxp@dlmu.edu.cn (X.K.); 2 State Key Laboratory of Coastal and Offshore Engineering, Dalian University of Technology, Dalian 116023, China; 3 Suzhou New District (Huqiu District) Shishan & Hengtang Sub-District Construction and Management Service Centre, Suzhou 215000, China; zhoulimin@dlmu.edu.cn; 4 Dalian Municipal Public Utilities Service Center, Dalian 116021, China; dodo_hong@126.com

**Keywords:** Liaodong Bay, dissolved heavy metals, seasonal variation, spatial distribution

## Abstract

Spatial–seasonal variations in dissolved heavy metals in surface seawater were analyzed based on surveys at 87 sampling sites and water samples from six rivers across Liaodong Bay. The concentrations of copper (Cu), lead (Pb), cadmium (Cd), and zinc (Zn) had ranges of 0.20–40.00 (5.45 ± 5.67), 0.51–33.64 (4.68 ± 3.93), 0.03–13.47 (2.22 ± 2.01), and 0.50–80.09 μg/L (14.22 ± 16.32), respectively, throughout the four seasons of 2020. The trace metal concentration showed a spatial gradient of high to low from river to estuary and from inshore to offshore areas. A combination of pollution levels and marine sensitivity was employed to assess the pollution degree of the heavy metals. As a whole, the single pollution factors of trace metals in Liaodong Bay were ranged in the order Pb > Zn > Cu > Cd. The total pollution degree was relatively high in autumn and summer due to increased riverine inputs after the rainy season, while relatively low in spring and winter. These findings provide baseline data for future targeting policies to protect marine environments in Liaodong Bay.

## 1. Introduction

Heavy metals in seawater have the characteristics of persistence, high toxicity and non-biodegradability, and bring adverse effects to marine ecology and human health through bioaccumulation. With the intensification of human activities, heavy metal pollution in estuaries and coastal waters has become increasingly serious.

Liaodong Bay is the largest semi-closed bay in the Bohai Sea of China, covering an area of approximately 10,000 km^2^. With the rapid increase in industrialization and economic development, various anthropogenic pollutants have been continuously discharged into this bay. Liaodong Bay receives a considerable portion of pollutants from several inflowing rivers including the Daliao River, Liao River, Daling River, Xiaoling River, Liugu River, and Fuzhou River, regarded as the main metal pollution sources for this bay [1]. Effluents from heavy industry bases nearby lead to high levels of heavy metals in parts of coastal waters [2]. Moreover, with the longest water exchange half-life, over two years, of the three main bays of the Bohai Sea, Liaodong Bay is incapable of cleaning itself once contaminated [3]. Extensive efforts have concentrated on the studies of heavy metals in the sediment of Liaodong Bay [4,5]. Mollusks show a considerable ability to accumulate heavy metals from seawater. Cd accumulation in oysters from Liaodong Bay poses a substantial human health risk [6]. Only a few studies have been conducted on the distribution of heavy metals in the seawater of Liaodong Bay (Wan, 2008), which focused on the distribution of heavy metals in water in summer, and the study area was restricted to the northern bay head [1].

There are four principal rivers inflowing into Liaodong Bay, with an annual runoff of 15 × 10^8^ m^3^. The seasonal natural runoff variation in these rivers is controlled by the East Asia Monsoon, the seasonal distribution of which is obviously uneven. The runoff in the wet season, lasting from June to October, accounts for more than 80% of the annual runoff. The variations in heavy metal concentrations between flood and dry season are not as obvious as runoff volume, and the annual distribution characteristics of different kinds of heavy metals are not synchronous. Therefore, the investigation and analysis of spatial distribution of and seasonal variations in heavy metals contributes to a comprehensive understanding of the pollution status and response mechanisms of seawater environments to natural and anthropogenic impacts.

## 2. Materials and Methods

**Figure 1:**
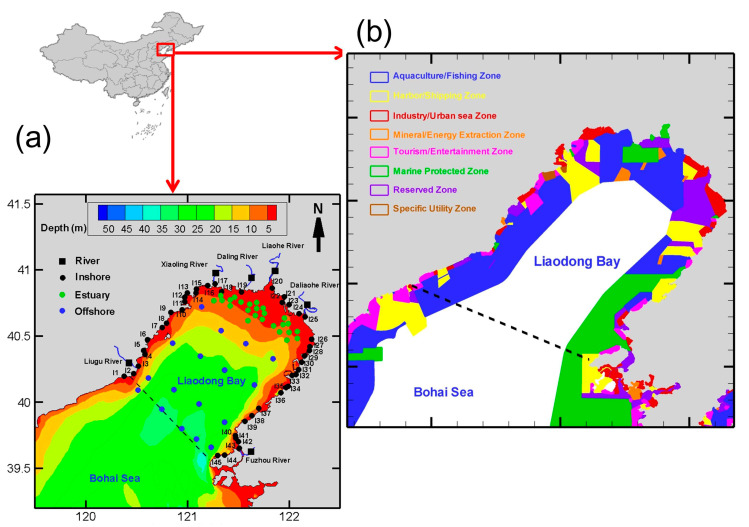
Sampling stations (**a**) and marine spatial planning areas (**b**) in the Liaodong Bay.

According to previous surveys, serious heavy metal pollution is not always found in estuaries but rather near coastal industrial areas, where abnormally high levels of certain heavy metals can be found. This fully demonstrates that coastal industrial emissions also make an important contribution to pollution in Liaodong Bay. Instead of previous studies focused on localized estuaries, ports, or industrial sea areas, we undertook a full cross-scale investigation of dissolved heavy metals from rivers to estuaries and from inshore to offshore areas. In this work, a total of 87 sampling sites were selected in Liaodong Bay and six sampling sites were selected closer to the mouth of major rivers flowing into this bay (Figure 1). Four sampling cruises were performed in February 2020 (winter), May 2020 (spring), August 2020 (summer), and October 2020 (autumn).

All analytical procedures were strictly monitored through quality assurance following the methods in GB17378.3 (2007) “The speciation for the marine monitoring—Part 3: Sample collection, storage and transportation” and GB17378.4 (2007) “The speciation for the marine monitoring—Part 4: Seawater analysis” [7,8]. Water was collected and immediately filtered through a 0.45 μm pore glass fiber filter into pre-cleaned polyethylene containers. The measurements of copper (Cu), lead (Pb) and cadmium (Cd) were carried out using absorption spectrometry (AAS), and concentration of zinc (Zn) was analyzed using graphite furnace atomic absorption spectrometry (GFAAS). The relative standard deviations for precision were 3.6%, 4.1%, 3.3% and 2.6% for Cu, Pb, Cd and Zn, respectively. Spike recoveries ranged from 86.2% to 107.5%.

The pollution factor (*PF*) is the ratio computed by dividing the concentration of each heavy metal in seawater (*C_i_*) by the background concentration (*C*_b_) (Equation (1)). The pollution degree (*PD*) is jointly determined by the sum of *PF* (2):

(1) $$PF_{i}=\frac{C_{i}}{C_{b}}$$

(2) $$PD=\sum_{i=1}^{N}PF_{i}$$

Due to the lack of heavy metal background concentrations previously determined in Liaodong Bay, concentrations at the grade seawater quality standard (GB3097—1997, 1997) were adopted as the background values [9]. Each marine functional area obeys specific water quality criteria; therefore, the pollution degree caused by identical concentrations in distinct areas may be different. Statistical data analyses were conducted with Microsoft Excel 2010, and spatial distributions of trace metals were drawn using Tecplot 360.

## 3. Results

### 3.1. Seasonal Variation in Dissolved Heavy Metals

**Figure 2:**
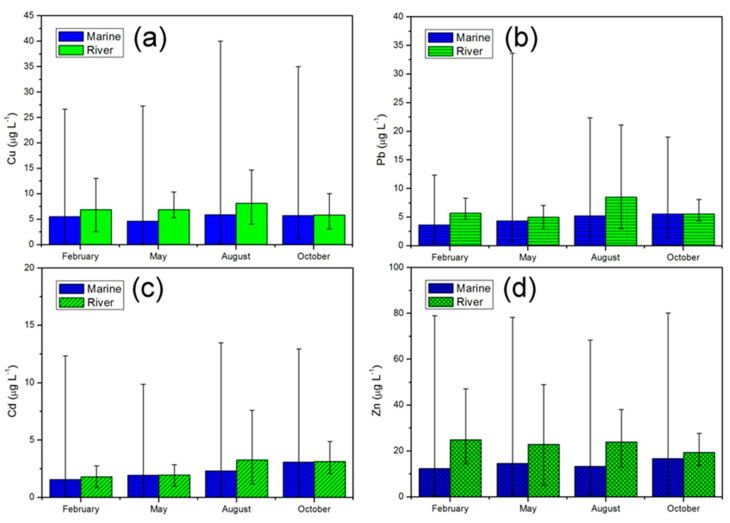
Metal seasonal variations in water samples from Liaodong Bay and rivers flowing into the bay. Error bars indicate standard errors. (**a**) Cu; (**b**) Pb; (**c**) Cd; (**d**) Zn.

Dissolved heavy metal concentrations in river water and surface seawater are presented in Figure 2. Concentrations of dissolved Cu, Pb, Cd, and Zn in seawater ranged 0.20–39.99, 0.50–33.64, 0.03–13.47, and 0.50–80.09 μg/L, respectively, following the order Zn > Cu > Pb > Cd by absolute value. Concentrations of dissolved Cu, Pb, Cd, and Zn in the rivers emptying into the bay ranged 3.11–14.66, 3.01–21.08, 0.85–7.59, and 13.67–49.04 μg/L, respectively. The concentration of dissolved heavy metals in Liaodong Bay varied greatly between stations, but the variation in heavy metals in the runoff into this bay maintained a narrow range. Although the concentration of heavy metals in seawater varied over a wider range, the average was greater in river water. Therefore, the river input could be recognized as an important source of heavy metals in Liaodong Bay.

Significant seasonal variations in dissolved heavy metals were found. The order of seasonal variation coefficients of dissolved heavy metals was Cd (0.293) > Pb (0.185) > Zn (0.130) > Cu (0.104). The highest seasonal mean Cu concentration appeared in summer, while Pb, Cd and Zn concentrations were elevated in autumn.

Linear correlation analysis was conducted to investigate the correlativity between the heavy metal concentrations in seawater and that in rivers emptying into Liaodong Bay. The correlation coefficients of Cu, Pb, Cd and Zn between seawater and river water were 0.198, 0.400, 0.817, and −0.898. Among the four measured metals, Cd was the highest in correlation coefficient, suggesting that runoff was the main pollution source. Unlike the other three metals, the correlation coefficient of Zn was close to −1. This indicated that municipal and industrial wastes from residential and industrial centers surrounding Liaodong Bay were important pollutant sources of Zn. The observation that the atmospheric deposition flow of Zn was higher than that via riverine discharge, put forward by Liang et al. (2018), supports our perspective [10].

**Figure 3:**
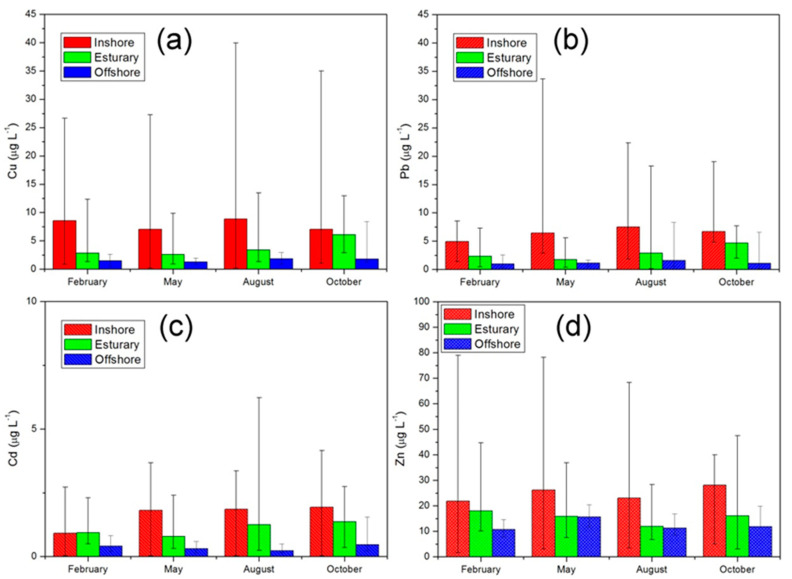
Seasonal and regional variations in Cu, Pb, Cd and Zn in seawater. Error bars indicate standard errors. (**a**) Cu; (**b**) Pb; (**c**) Cd; (**d**) Zn.

In order to understand the spatial distribution characteristics, the 87 sampling sites were divided into three groups: 45 located in the inshore area, 25 located in the estuary near the bay head, and 17 in the offshore area. The range and mean concentrations of dissolved Cu, Pb, Cd and Zn at sub-regional sampling stations are illustrated in Figure 3. It was found that the four dissolved heavy metal concentrations varied both seasonally and spatially. All the metal concentrations had a similar distribution pattern, following the order inshore > estuary > offshore.

The potential sources of dissolved heavy metals in seawater include: (1) river input; (2) human activities; (3) weathering and erosion of coastal rocks and soils; (4) atmospheric deposition [11]. Overall, the fluctuation range of heavy metal concentration in the inshore area was the largest, and the mean value was the highest. This is primarily due to coastal regions having high population density and intense human activity. A large number of manufacturing companies and metal smelting plants are located along the coast of Liaodong Bay. In some cases, industrial wastewater not treated or not up to standards is directly discharged into coastal areas, which could lead to serious heavy metal contamination. Atmospheric inputs play a role in the supply of heavy metals to the sea, especially important in semi-enclosed seas such as Liaodong Bay, which is close to potential pollution sources in industrial and urban areas and subjected to the well-known inflow of East Asian dust storms [12]. Various sources, together with shallow water depth and poor water dilution capacity, easily lead to an increase in pollutant concentration.

The mean annual runoff flows of the Daliao River, Liao River, Daling River, Xiaoling River, Liugu River and Fuzhou River are 77.15 × 10^8^ m^3^, 44.37 × 10^8^ m^3^, 17.91 × 10^8^ m^3^, 3.98 × 10^8^ m^3^, 5.37 × 10^8^ m^3^, and 3.32 × 10^8^ m^3^. Despite a lower annual flow of dissolved trace metals from rivers into the head of Liaodong Bay, concentrations of dissolved heavy metals continually increase due to low exchange quantity of water between the bay and the open sea.

The seasonal average concentrations of Cu, Pb, Cd and Zn in the estuary area were 3.72, 2.91, 1.08 and 15.50 μg/L, respectively. All four types of dissolved trace metals were at higher concentrations than those in other large estuaries, such as the Yangtze River Estuary [13], Yellow River Estuary [14], and Pearl River Estuary [15].

Two distinct rainfall-based seasons in the Liao River Basin were normally identified, including the rainy season (from June to September) and the dry season (from October to May). The rainy season causes soil to erode and thus brings eroded sediment and agricultural runoff into rivers. Among these rivers, the Daliao River and Liao River are the top two by runoff amount. Flowing through highly developed and industrialized areas, they receive large amounts of heavy metals from both natural and anthropogenic origins and become important sources for Liaodong Bay. In the flood season, riverine discharge dominates the input of all four metals into the head of Liaodong Bay. Nevertheless, the lower concentration of Zn near the Liaohe Estuary compared to other areas is attributed to high sedimentation rates and dilution by increased freshwater with low input concentration.

The Cu concentration of inshore samples in the summer and winter seasons were slightly higher than in the spring and autumn seasons. The mean concentration of Cu in the estuarine area was highest in autumn, indicating that influx of water from the surrounding rivers was the most likely source in the wet season.

The highest concentration of Pb in the estuary area appeared in October, meaning that river inputs in the estuary area were the primary source during the wet season. The maximum concentration in the inshore zone occurred in August due to the large discharge along the coast, in accordance with the peak summer travel season.

Based on previous studies, it is generally believed that the concentration of Pb in winter in the waters of Liaodong Bay is relatively low [16]. Our findings generally support this conclusion. Heavy metal concentrations at the winter inshore stations were significantly lower compared to those of the other three seasons, mainly due to the particularity of our survey time, when epidemic control measures were implemented in China such as traffic control and social distancing. Sharply reduced human activities resulted in a significant decline in emissions, and this effect is particularly obvious for lead emissions from vehicle exhaust. Concentrations increased significantly in the inshore area in May as social activity resumed.

As rivers were the main sources of this metal, concentrations of Cd in the estuary areas in summer and autumn were significantly higher than those in winter and spring. Meanwhile, several rivers with large differences in Cd concentrations flow into the sea at the head of the bay, resulting in a great variation in range among stations in the estuary areas.

The spatial distribution of Zn was relatively uniform. Although the concentrations of Zn were highest at the inshore stations, the differences from other regional sites were less significant than those of the other three metals. Liang et al. (2018) argued that the atmospheric deposition flow of Zn into Liaodong Bay was even higher than that via river discharge [10].

### 3.2. Spatial Distributions of Dissolved Heavy Metals

**Figure 4:**
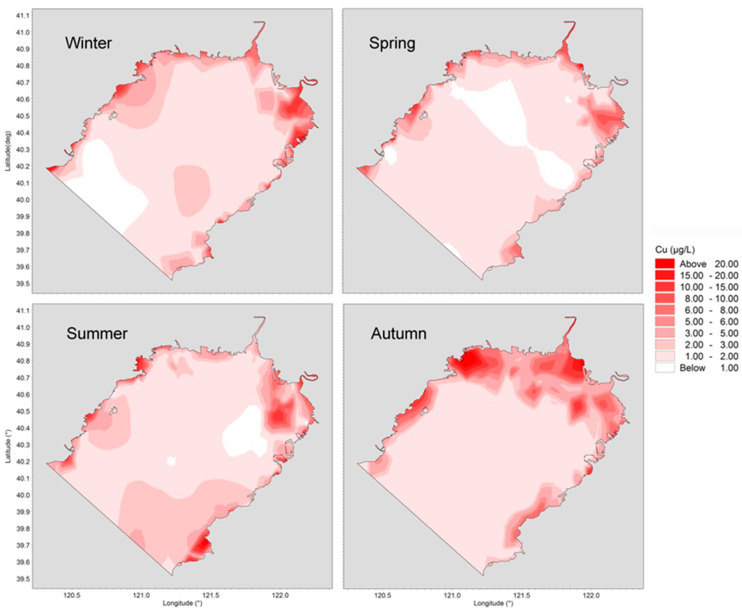
Horizontal distributions of dissolved Cu in Liaodong Bay from February 2020 to October 2020.

**Figure 5:**
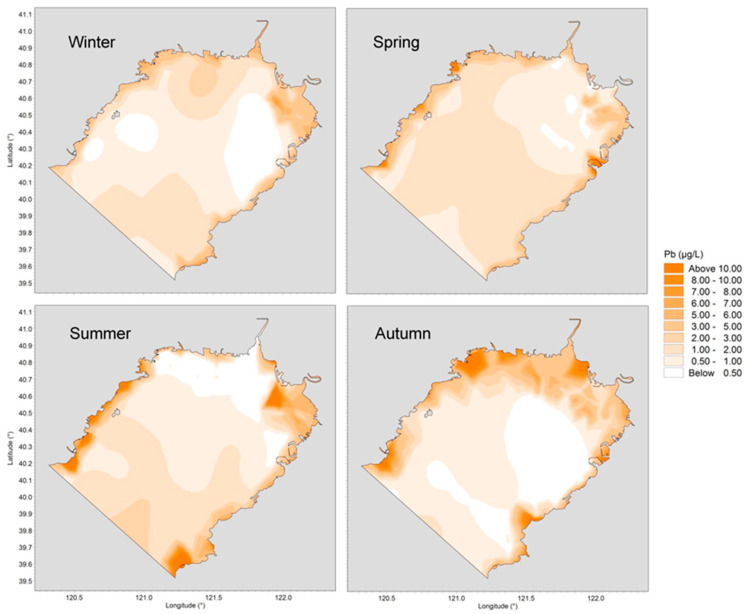
Horizontal distributions of dissolved Pb in Liaodong Bay from February 2020 to October 2020.

**Figure 6:**
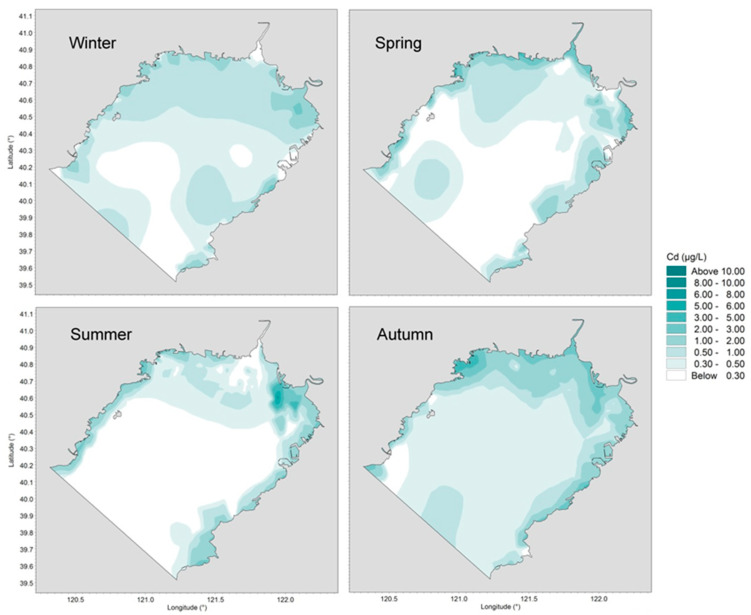
Horizontal distributions of dissolved Cd in Liaodong Bay from February 2020 to October 2020.

**Figure 7:**
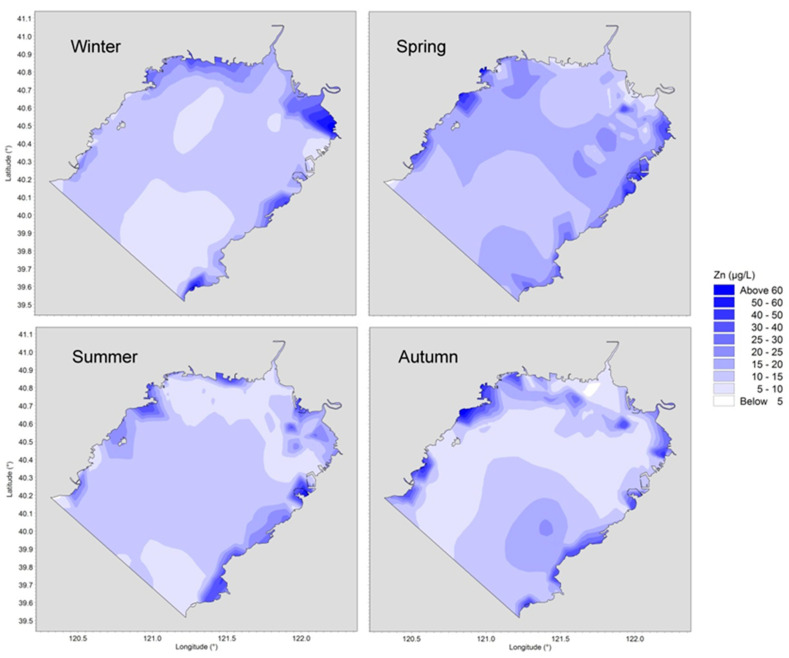
Horizontal distributions of dissolved Zn in Liaodong Bay from February 2020 to October 2020.

The behavior and fate of trace metals are determined by their own physicochemical properties as well as by environmental conditions such as water temperature, pH, salinity, current circulation structures, sediment concentration, etc. Horizontal distributions of concentrations of dissolved Cu, Pb, Cd and Zn in four different seasons are presented in Figure 4, Figure 5, Figure 6 and Figure 7.

In winter, the distribution of Cu was relatively uniform due to the strong north wind-induced dispersion effect. A high-level area appeared from Daliaohe Estuary to Bayuquan Port, where contaminants accumulate under the action of seasonal circulation. The level of Cu was low in spring, and the concentration in most sea areas was less than 3 μ g/L due to a long low-input season of half a year. The spatial distribution was similar in summer and spring, with high levels in the sea west of Yingkou. After the wet season, the concentration of Cu increased to up to 5.91 μg/L, due to large loads in river runoff.

As shown in Figure 5, Pb concentration was relatively high in summer and autumn, and was lowest in winter. The spatial patterns were similar in winter and spring, and the distribution was even in the offshore waters. In summer, the distribution trend was high on the east and west coast and low in the offshore area. Coastal industry sewage discharge and anthropogenic emissions greatly affected Liaodong Bay in the summer, and the extent of Pb pollution in the inshore area was apparently higher than in the offshore area. During autumn, riverine discharge dominated the input of Pb into the marine environment, causing high concentrations near the northern bay head and lower ones at the broad area around the outlet of the bay.

Cd concentration in autumn was evidently higher than in the other seasons. This was the result of increased metal concentration in river runoff after the wet season. The riverine Cd load in coastal waters was concentrated at the head of the bay and dispersed to the whole bay throughout the autumn due to the action of northerly wind. Due to continuous low riverine Cd exports to coastal waters, the levels decreased continuously from winter to summer in open seas less affected by runoff. Cd concentration in Jinzhou Bay, a sub-bay located in the northwest corner of Liaodong Bay, maintained over 1 μg/L all year round, because of local long-term industrial sewage discharge.

As shown in Figure 7, high concentrations of Zn appeared along both sides of the Liaodong Bay and low ones in the offshore area. The concentration in the open offshore was basically between 10–20 μg/L, while the average value near the inshore was not less than 20 μg/L in any season. Unlike the other three metals, the concentration of Zn at the head of the bay was lower than that in other areas. The primary reason was that the concentration of runoff into the water was not high and the large amount of river water volume played a diluting role during the wet season.

## 4. Discussion

**Figure 8:**
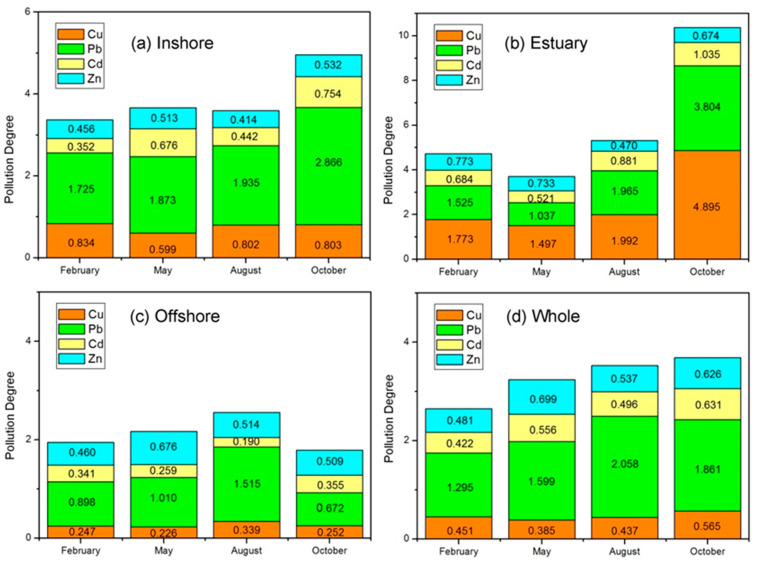
Pollution factors for dissolved heavy metals in different regions of Liaodong Bay at different months.

Pollution factors for dissolved heavy metals (Cu, Pb, Cd and Zn) in different regions of Liaodong Bay and for different months are presented in Figure 8. As a whole, the pollution level of Liaodong Bay was low, with *PD* values ranging from 2.649 to 3.684, according to the recommended value standards [17]. However, the regional diversity of heavy metal pollution in Liaodong Bay is relatively obvious.

We identified the estuary at the head of Liaodong Bay as the most contaminated subarea with *PD* values ranging from 3.704 to 10.372. In autumn, pollution reached considerable levels with *PD* exceeding 10. The inshore pollution was second only to the estuary, which reflects that coastal discharge is also an important input source of heavy metals in Liaodong Bay. The stable emission intensity led to relatively slight seasonal changes. Due to strong dilution capacity and the long distance from the pollution sources, *PD* of the offshore area was less than 3.0 in any season, which presents a low pollution level with respect to heavy metals.

Among the four metals, Pb was almost always the dominant pollutant at any scale of Liaodong Bay. A large amount of discharge from land was the dominant factor. It is estimated that 55.7% of the Pb flowing from terrestrial sources into the Bohai Sea is injected into Liaodong Bay [18]. Although the absolute concentration of Zn was highest, its relative pollution degree was second to Pb because of its smaller values relative to the water quality standard. The high-risk area for Cu was concentrated near the estuary, where it even surpassed Pb as the primary contaminant in October, and the seasonal fluctuation was evident with a seasonal variation coefficient up to 0.619. The pollution degree of Cd in Liaodong Bay was relatively low, and it decreased obviously in the seaward direction. Cd concentrations in coastal oysters exceeded national safety guidelines, indicating trace metal pollution seriousness in the inshore zone.

**Figure 9:**
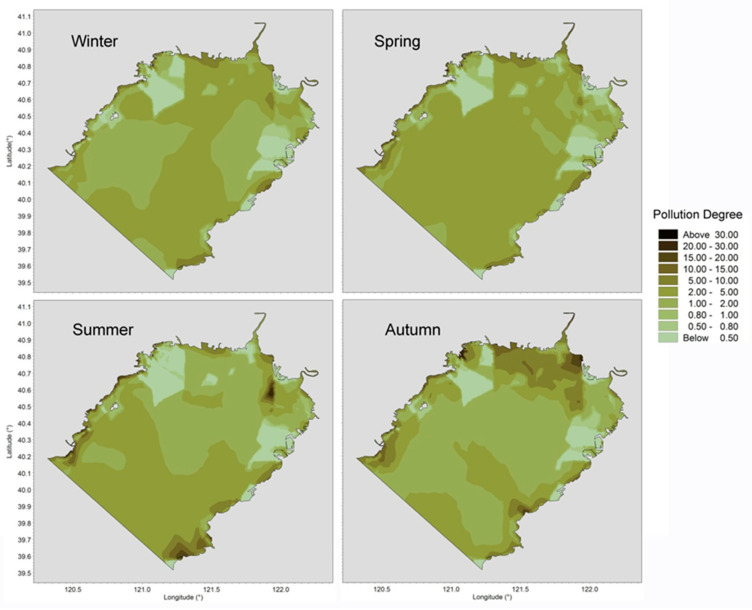
Horizontal distributions of dissolved heavy metal pollution degree in Liaodong Bay from February 2020 to October 2020.

By interpolating measurements onto grid points, the horizontal pollution degree distributions in different months are depicted in Figure 9. It should be noted that areas with high concentrations are not necessarily areas with higher levels of pollution. The concentrations of heavy metals in the waters near Jinzhou Port and Yingkou Port were high, while the pollution degree was relatively low due to lower water quality requirements for harbor/shipping zones. By contrast, the marine conservation area near Changxing Island suffered from a higher pollution degree due to its stricter requirements for water quality. Monitoring and analyzing the results of our study would be useful in exploiting ocean resources in order to minimize conflicts between economic benefits and environmental protection.

Our study confirms that heavy metal concentrations in seawater were highest in autumn, whereas the previous survey found that the most severe contamination occurred in summer [6]. Inter-annual differences are normal, due to changes in flood season duration and rain intensity. This highlights the need for long-term follow-up monitoring. Our observations from 87 sampling sites seem more reliable than the results of Liu et al. (2021), whose just five sample sites were insufficient to produce credible data reflecting seasonal variability. Moreover, all of Liu’s sampling sites were located on the coast, and could not characterize the pollution status in offshore areas. A large-scale investigation focused on trace metals in marine fish from Liaodong Bay identified high concentrations of Pb in Cynoglossus joyneri and Zn in Hemirhamphus sajori [19]. These previous studies on marine wild fish are consistent with our assessments on the risk degree of different metals.

## 5. Conclusions

To study the seasonal variations in and spatial distributions of dissolved heavy metals in Liaodong Bay, field observations of copper, lead, cadmium, and zinc at 93 stations (87 within the bay and six at its inflowing rivers) were conducted in all four seasons of 2020. In terms of absolute magnitude, the concentration of Zn was the highest among the four heavy metals, but in terms of relative contamination degree, Pb was regarded as the primary pollutant. Variations in the four metals across different seasons were significant due to both input intensity and hydrodynamic factors.

The most recent survey results are beneficial for marine spatial planning and environmental remediation. Further biomonitoring study is needed to assess the bioavailability of heavy elements in different marine organisms to understand bioaccumulation patterns and evaluate their potential ecological risk.

## Data Availability

The data presented in this study are available on request from the corresponding author.
